# Supplementary material for: Genetic, cellular, and structural characterization of the membrane potential-dependent cell-penetrating peptide translocation pore
Source: eLife. 2021 Oct 29;10:e69832. doi: 10.7554/eLife.69832 (PMC8639150; doi:10.7554/eLife.69832)
Supplement: Supplementary file 1. — This table lists the components found in the Biowest RPMI-like media that lacks potassium chloride and sodium chloride. [file elife-69832-supp1.docx]

**Supplementary File 1**

| **CAS Number** | **Components** | **Quantity in g/l** |
| --- | --- | --- |
| 13477-34-4 | Calcium Nitrate Tetrahydrate | 0.10000000 |
| 7487-88-9 | Magnesium Sulfate Anhydrous | 0.04884000 |
| 50-99-7 | D-Glucose Anhydrous | 2.00000000 |
| 56-40-6 | Glycine | 0.01000000 |
| 39537-23-0 | L-Alanyl-L-Glutamine | 0.44600000 |
| 74-79-3 | L-Arginine Free Base | 0.20000000 |
| 70-47-3 | L-Asparagine Anhydrous | 0.05000000 |
| 56-84-8 | L-Aspartic acid | 0.02000000 |
| 30925-07-6 | L-Cystine Dihydrochloride | 0.06520000 |
| 56-86-0 | L-Glutamic Acid | 0.02000000 |
| 71-00-1 | L-Histidine | 0.01500000 |
| 51-35-4 | L-Hydroxy-L-Proline | 0.02000000 |
| 73-32-5 | L-Isoleucine | 0.05000000 |
| 61-90-5 | L-Leucine | 0.05000000 |
| 657-27-2 | L-Lysine Monohydrochloride | 0.04000000 |
| 63-68-3 | L-Methionine | 0.01500000 |
| 63-91-2 | L-Phenylalanine | 0.01500000 |
| 147-85-3 | L-Proline | 0.02000000 |
| 56-45-1 | L-Serine | 0.03000000 |
| 72-19-5 | L-Threonine | 0.02000000 |
| 73-22-3 | L-Tryptophan | 0.00500000 |
| 69847-45-6 | L-Tyrosine Disodium Salt Dihydrate | 0.02883000 |
| 72-18-4 | L-Valine | 0.02000000 |
| 67-48-1 | Choline Chloride | 0.00300000 |
| 58-85-5 | D-Biotin | 0.00020000 |
| 137-08-6 | D-Ca Pantothenate | 0.00025000 |
| 59-30-3 | Folic Acid | 0.00100000 |
| 87-89-8 | Myo-Inositol | 0.03500000 |
| 98-92-0 | Nicotinamide (Nicotinic acid amide) | 0.00100000 |
| 150-13-0 | P-Aminobenzoic Acid (PABA) | 0.00100000 |
| 58-56-0 | Pyridoxine Hydrochloride | 0.00100000 |
| 83-88-5 | Riboflavin | 0.00020000 |
| 67-03-8 | Thiamine Hydrochloride | 0.00100000 |
| 68-19-9 | Vitamine B12 | 0.00000500 |
| 70-18-8 | L-Glutathione Reduced | 0.00100000 |
| 34487-61-1 | Phenol Red Sodium Salt | 0.00530000 |
| WATER |  | 996.66117500 |
