# Supplementary material for: Genetic, cellular, and structural characterization of the membrane potential-dependent cell-penetrating peptide translocation pore
Source: eLife. 2021 Oct 29;10:e69832. doi: 10.7554/eLife.69832 (PMC8639150; doi:10.7554/eLife.69832)
Supplement: Supplementary file 2. — This table lists the proportions of various lipids found in the inner and outer layers of the plasma membrane that we have used for our simulations. [file elife-69832-supp2.docx]

**Supplementary file 2**

|  | **Inner membrane** | | **Outer membrane** | |
| --- | --- | --- | --- | --- |
| type | Number of lipids | % | Number of lipids | % |
| POPC | 18 | 18 | 39 | 39 |
| POPE | 27 | 27 | 6 | 6 |
| PSM | 10 | 10 | 21 | 21 |
| POPS | 11 | 11 | - | - |
| POPI | 5 | 5 | - | - |
| CHOL | 29 | 29 | 34 | 34 |
| total | 100 | 100 | 100 | 100 |
