# Supplementary material for: Genetic, cellular, and structural characterization of the membrane potential-dependent cell-penetrating peptide translocation pore
Source: eLife. 2021 Oct 29;10:e69832. doi: 10.7554/eLife.69832 (PMC8639150; doi:10.7554/eLife.69832)
Supplement: Supplementary file 3. — This table reports the free energy that needs to be overcome for the formation of water pores that was calculated in the indicated studies. [file elife-69832-supp3.docx]

**Supplementary file 3**

| **Force field** | **Free energy difference (kJ/mol)** | **References** |
| --- | --- | --- |
| All-atom (CHARMM36) | 95.4 | Ting, C.L. et al.^2^ |
| All-atom  (CHARMM36) | 104.6 (by extrapolation) | Dixit, M. et al.^1^ |
| CG-MARTINI | 93+/-1.5 | This study |

1 Dixit, M. & Lazaridis, T. Free energy of hydrophilic and hydrophobic pores in lipid bilayers by free energy perturbation of a restraint. *J Chem Phys* **153**, 054101, doi:10.1063/5.0016682 (2020).

2 Ting, C. L., Awasthi, N., Muller, M. & Hub, J. S. Metastable Prepores in Tension-Free Lipid Bilayers. *Phys Rev Lett* **120**, 128103, doi:10.1103/PhysRevLett.120.128103 (2018).
