# Supplementary material for: Genetic, cellular, and structural characterization of the membrane potential-dependent cell-penetrating peptide translocation pore
Source: eLife. 2021 Oct 29;10:e69832. doi: 10.7554/eLife.69832 (PMC8639150; doi:10.7554/eLife.69832)
Supplement: Supplementary file 4. — The green nucleotides correspond to the bar codes. The red nucleotides are added to increase library complexity to prevent signal saturation when the sequencing is performed. The blue sequences are complementary to the extremities of the first PCR fragments. [file elife-69832-supp4.docx]

**Supplementary file 4**

| F2a | AATGATACGGCGACCACCGAGATCTACACTCTTTCCCTACACGACGCTCTTCCGATCTTCTTGTGGAAAGGACGAAACACCG |
| --- | --- |
| F2b | AATGATACGGCGACCACCGAGATCTACACTCTTTCCCTACACGACGCTCTTCCGATCTAGCTCTTGTGGAAAGGACGAAACACCG |
| F2c | AATGATACGGCGACCACCGAGATCTACACTCTTTCCCTACACGACGCTCTTCCGATCTCGAGCTCTTGTGGAAAGGACGAAACACCG |
| F2d | AATGATACGGCGACCACCGAGATCTACACTCTTTCCCTACACGACGCTCTTCCGATCTCATAACCTCTTGTGGAAAGGACGAAACACCG |
| F2e | AATGATACGGCGACCACCGAGATCTACACTCTTTCCCTACACGACGCTCTTCCGATCTGTGCTAACGTCTTGTGGAAAGGACGAAACACCG |
| R2_iC_26 | CAAGCAGAAGACGGCATACGAGAT**GCTCAT**GTGACTGGAGTTCAGACGTGTGCTCTTCCGATCTTCTACTATTCTTTCCCCTGCACTGT |
| R2_iA_12 | CAAGCAGAAGACGGCATACGAGAT**TACAAG**GTGACTGGAGTTCAGACGTGTGCTCTTCCGATCTTCTACTATTCTTTCCCCTGCACTGT |
