# Supplementary material for: Genetic, cellular, and structural characterization of the membrane potential-dependent cell-penetrating peptide translocation pore
Source: eLife. 2021 Oct 29;10:e69832. doi: 10.7554/eLife.69832 (PMC8639150; doi:10.7554/eLife.69832)
Supplement: Supplementary file 5. — This table provides the sequences of the sgRNAs used to target the first exon of the indicated genes. [file elife-69832-supp5.docx]

**Supplementary file 5**

| **Target gene** | **sgRNA name** | **sgRNA sequence** |
| --- | --- | --- |
| KCNN4 | sgKCNN4.1 | CTGCCCGAGTGCTACAAGAA |
| KCNN4 | sgKCNN4.2 | CATGGTGCCCGGCACCACGT |
| KCNK5 | sgKCNK5.1 | ATGGTGGTAATGACGGTCGC |
| KCNK5 | sgKCNK5.2 | CTCTGCCTGACGTGGATCAG |
| KCNQ5 | sgKCNQ5.1 | TCTAGGAATTAATTCACAGC |
| KCNQ5 | sgKCNQ5.2 | ACAGATCCTCCGCATGGTCG |
