# Supplementary material for: Genetic, cellular, and structural characterization of the membrane potential-dependent cell-penetrating peptide translocation pore
Source: eLife. 2021 Oct 29;10:e69832. doi: 10.7554/eLife.69832 (PMC8639150; doi:10.7554/eLife.69832)
Supplement: Supplementary file 6. [file elife-69832-supp6.docx]

**Supplementary file 6**

| **Target gene** | **Oligo name** | **Oligo sequence** |
| --- | --- | --- |
| KCNN4 | KCNN4.1.TA.F | GCAGAGAAGCACGTGCA |
| KCNN4 | KCNN4.1.TA.R | GGCAGCATGAGACTCCTTCC |
| KCNQ5 | KCNQ5.1.TA.F | GGGACATGATGTACAATGGA |
| KCNQ5 | KCNQ5.1.TA.F | CCAGAGAGCATCTGCATATG |
